# The clinical utility of a comprehensive psychosomatic assessment in the program for colorectal cancer prevention: a cross-sectional study

**DOI:** 10.1038/s41598-021-95171-8

**Published:** 2021-08-02

**Authors:** Sara Gostoli, Maria Montecchiarini, Alessia Urgese, Francesco Ferrara, Anna Maria Polifemo, Liza Ceroni, Asia Gasparri, Chiara Rafanelli, Vincenzo Cennamo

**Affiliations:** 1 Department of Psychology, University of Bologna, Viale Berti Pichat 5, 40127, Bologna, Italy; 2 Gastroenterology and Interventional Endoscopy Unit, AUSL Bologna, Bologna, Italy

**Keywords:** Cancer prevention, Cancer screening, Gastrointestinal cancer, Cancer, Psychology, Risk factors

## Abstract

Few studies have investigated psychosocial characteristics and lifestyle behaviors of participants at programs for secondary prevention of colorectal cancer (CRC). This study aimed, through a comprehensive psychosomatic assessment based on clinimetric principles, to evaluate psychosocial characteristics and lifestyle behaviors in participants at CRC secondary prevention program, and to investigate the associations between these variables and endoscopic outcomes. In this cross-sectional study, the first 150 consecutive asymptomatic participants at the CRC prevention program who resulted positive to fecal occult blood test (FOBT) and were thus referred to colonoscopy, underwent a psychosomatic assessment including psychiatric diagnoses (DSM-5), psychosomatic syndromes (DCPR-R), psychological distress, psychological well-being and lifestyle behaviors. Whereas only 5.3% of the sample showed at least one DSM-5 diagnosis, 51.3% showed at least one DCPR syndrome, such as allostatic overload, alexithymia, Type A behavior, and demoralization. Patients affected by psychosomatic syndromes presented with significantly higher psychological distress, lower psychological well-being and unhealthy lifestyle behaviors, such as tobacco smoking and unhealthy diet, in comparison with patients without DCPR syndromes. Among endoscopic outcomes, the presence of adenomas was significantly associated with DCPR irritable mood. In a clinical context of secondary prevention addressing asymptomatic patients with positive FOBT, a comprehensive psychosomatic assessment may provide relevant clinical information for those patients who present certain psychosomatic syndromes associated with high psychological distress, impaired psychological well-being, unhealthy lifestyle behaviors and colorectal precancerous lesions. The results of the present study indicate a road to the practice of “preventive” medicine at CRC screening program.

Colorectal cancer (CRC) is the third most deadly and fourth most common form of cancer in the world^1^. Its incidence has been steadily rising worldwide, especially in countries undergoing a major economic transition, which are adopting the “western” way of life^2^. Obesity, sedentary lifestyle, red/processed meat consumption, alcohol, and tobacco smoking are considered the leading factors behind the growth of CRC^1,3^. On the contrary, healthy lifestyle, physical activity, daily consumption of fibers and dairy products are inversely associated with the development of CRC^3^. Recent advances in early detection screenings and treatment options have reduced CRC incidence and mortality in developed countries^2,4^. However, cases of CRC appearing at a younger age have increased significantly in recent years in the US and Europe^2,5^. In addition, CRC still represents the third leading cause of cancer-related deaths for both men and women in Italy^6,7^.

Adherence to a healthy lifestyle is associated with a reduced risk of colorectal cancer regardless of individuals’ genetic risk^8^. It has been found that a healthy lifestyle is strongly associated with lower risk of all stages of colorectal neoplasms^9^. Erben et al.^9^ highlighted the importance of a healthy lifestyle early in the beginning of the carcinogenic process and strengthened its relevance for primary prevention purposes. However, Ladabaum, et al.^4^ reported difficulties in implementing major lifestyle changes or widespread primary prevention strategies to decrease CRC risk. Moreover, recent findings suggested that psychosocial distress might moderate the modification of specific health-related behaviors (such as physical activity, behavioral aspects of food consumption, stress management and pharmacological adherence) in cardiac rehabilitation^10^. Nevertheless, few studies have investigated the psychosocial characteristics of participants at programs for secondary prevention of CRC, and most of them aimed at assessing levels of acute distress^11,12^ and quality of life^13^ only in view of the colonoscopy, and few at assessing personality^14^. In particular, Lauriola et al.^14^ found that patients with both adenoma and adenocarcinoma showed higher TAS-20 alexithymia scores, concerning the difficulty identifying feelings and externally oriented thinking, in comparison with patients with negative endoscopic outcomes. Among the limitations of their study, Lauriola et al.^14^ underlined the use of a single self-reported measure of alexithymia, despite the fact that in literature it had been recommended a multi-method, multi-measure approach for cross validating the research findings as well as to highlight whether or not different processes may relate alexithymia to health. The Authors also highlighted the need to assess a large set of mediators (such as mood states and depression) required to investigate which psychosocial or medical factors actually provide the link between disordered affect regulation and colon cancer. In the same vein, Sales et al.^15^, advocated the clinical utility of conducting a comprehensive psychosomatic evaluation in CRC patients including personality, as they found that Type-D (distressed) personality may predict distress among CRC patients and other personality traits may influence coping responses and quality of life in patients with CRC.

A considerable body of evidence has accumulated in psychosomatic medicine related to concepts such as stressful life events, illness behavior and personality. The comprehensive psychosomatic assessment proposed by Fava et al.^16^ allows routine evaluations of psychosocial factors according to clinimetric principles^17^ and may represent a crucial step toward the application of individualized care and effective patient management. Among psychosocial factors affecting individual vulnerability of any type of disease, Fava et al.^16^ include life events and allostatic load, health attitudes and behavior, psychological well-being and personality. Among psychological factors affecting course and outcome of a disease, the authors encompass patient-reported distress, illness behavior, demoralization and irritable mood, and psychiatric disorders.

Given the paucity of data on psychosocial characteristics affecting both vulnerability and course of disease in patients at secondary prevention of CRC, we wonder if such a comprehensive psychosomatic assessment could detect subgroups of patients presenting psychosocial factors, at higher risk for unhealthy lifestyle behaviors and worst endoscopic outcomes after the colonoscopy.

The aims of the present observational study are to evaluate: (1) psychological distress, well-being and lifestyle behaviors, through a comprehensive psychosomatic assessment^16^, in participants to CRC screening, promoted by the National Health System, who had a positive fecal occult blood test (FOBT) and who had been referred to colonoscopy; (2) the associations between psychosocial characteristics and lifestyle-related behaviors; (3) the associations of psychosocial characteristics and lifestyle behaviors with major endoscopic outcomes (i.e., precancerous lesions).

## Methods

### Participants

A 2-step approach community based CRC screening program is directed to men and women from 50 to 69 years old, referred to undergo fecal occult blood test (FOBT) every other year, and subsequent colonoscopy if FOBT is positive^18^. Thus, participants at this CRC screening prevention program who resulted positive at FOBT, were contacted during the study period (January 2019–June 2019) by the Regional Screening Centre and scheduled an appointment at Bellaria Hospital in Bologna (Italy) for an interview with a nurse the week before the colonoscopy, to give them instructions on how to prepare themselves for the exam (i.e. what they should not eat/drink before the colonoscopy and which medications they should take the day before).

### Procedure

Patients were asked to join the present study after the end of the nurse-interview. The first 150 consecutive FOBT-positive subjects, who accepted to undergo the psychological interview, were enrolled in the study. The ethic committee of the local health authority (AUSL Bologna, Italy) approved the study (Ref: 530/2018/OSS/AUSLBO). The research has been conducted according to the guidelines of the World Medical Association Declaration of Helsinki. All the participants provided written informed consent. Patients were excluded if they did not give their written informed consent to join the study or if they previously received a diagnosis of psychotic disorder.

### Assessment

After participants’ sociodemographic data on sex, age, employment, marital status and their previous adherence to CRC screening were collected, patients were interviewed by a clinical psychologist, according to a comprehensive psychosomatic assessment^16^. The participants thus underwent three validated clinical interviews (SCID-5, DCPR-R, and Psychological Well-Being Interview—PWB-I) and completed a self-rating questionnaire (SQ). The clinical psychologist also detected patients’ lifestyle habits (physical activity, dietary habits, alcohol consumption and tobacco smoking). The psychological assessment lasted about thirty minutes.

#### Psychiatric diagnoses

The Italian translation^19^ of the Structured Clinical Interview for DSM-5, clinician version (SCID-5-CV)^20,21^, were used in order to identify major depression, anxiety disorders (panic disorder, generalized anxiety disorder, agoraphobia, social anxiety), eating disorders (bulimia, binge eating disorder, anorexia nervosa), obsessive–compulsive disorder, somatic symptoms and related disorders (somatic symptoms disorders; illness anxiety). The SCID-5-CV showed excellent reliability, high specificity and clinical validity, which supports its use in daily clinical practice^22,23^.

#### Psychosomatic syndromes

The Italian version of the Semi-Structured Interview based on the revised version of the Diagnostic Criteria for Psychosomatic Research (DCPR-R)^16^ was used to identify psychosomatic syndromes. The updated version of DCPR was developed based on insights derived from their use in a large number of patients and settings^24,25^ and it includes the diagnostic criteria for two additional syndromes, allostatic overload and hypochondriasis. Both allostatic overload and hypochondriasis can be assessed by specific clinimetric criteria^16,26,27^ that underwent validation^27–31^. The interview based on DCPR-R has been used already in medical setting^32^ and it consists of dichotomous (i.e., yes or no) items and skip instructions. It allows assessing the presence of 14 psychosomatic syndromes divided into 4 clusters: stress, personality, illness behavior and psychological manifestations^16^. The first cluster includes allostatic overload (characterized by the presence of a current identifiable stressor in the form of recent life event or chronic stress exceeding the individual coping skills). The cluster of personality includes two syndromes that can potentially affect general vulnerability to disease, such as type A behavior (characterized by high competitiveness, excessive degree of involvement in work and other activities subject to deadlines, tendency to speed up mental and physical activities) and alexithymia (represented by the inability to use appropriate words to describe emotions). Illness behavior refers to the ways in which given symptoms may be differentially perceived, evaluated, and acted (or not acted) by different kinds of persons. The clinical spectrum of illness behavior encompasses eight syndromes according to DCPR-R criteria: hypochondriasis (i.e., persistent fears of having, or the idea of having, a serious disease based on misinterpretation of bodily symptoms); disease phobia (i.e., persistent, unfounded fear of suffering from a specific disease); thanatophobia (i.e., sense of impending death and/or conviction of dying soon); health anxiety (i.e., generic worry about illness, concern about pain, and bodily preoccupations); persistent somatization (i.e., functional medical syndromes such as fibromyalgia or chronic fatigue that cause distress and seeking medical care, and result in impaired quality of life); conversion symptoms (i.e., one or more symptoms or deficits affecting voluntary motor or sensory function characterized by lack of anatomical or physiological plausibility); anniversary reaction (i.e., symptoms of autonomic arousal occurring at the anniversary of specific negative events); illness denial (i.e., persistent denial of having a physical disorder that needs treatment). The cluster of psychological manifestations includes: demoralization (i.e., a feeling state characterized by the perception of being unable to cope with some pressing problems); irritable mood (i.e., a feeling state characterized by frequent manifestations of irritability that lack of their cathartic effect) and somatic symptoms secondary to a psychiatric disorder (i.e., somatic symptoms occurring after a psychiatric disorders that cause distress and impaired quality of life)^16^. The DCPR-R allow the identification of psychosocial conditions often neglected by traditional nosography. These criteria have been developed with the intent to operationalize the spectrum of manifestations of illness behavior and sub-threshold distress in both psychiatric and medical settings, and can be used independently of and in addition to the DSM criteria^27^. The use of DCPR was reported to be useful and reliable in the assessment and description of psychosomatic distress in general, medical and psychiatric populations, showing excellent interrater reliability, construct validity and predictive validity for psychosocial functioning and treatment outcome^32,33^.

#### Distress

The Italian version of Kellner’s Symptom Questionnaire (SQ)^34,35^ was used in order to identify subclinical psychological distress. It is a 92-item dichotomous self-rating scale, including items that may be rated as ‘yes’/‘true’ or ‘no’/‘false’. It yields four total scales (anxiety, depression, somatization and hostility-irritability) divided into four sub-scales of well-being (relaxation, contentment, physical well-being and friendliness) and four sub-scales of distress (symptoms of anxiety, depression, somatization and hostility-irritability). The score of each total scale may range from 0 (no symptoms) to a maximum of 23 (all the symptoms are present). The scale has been validated in several languages and used in numerous studies among various age populations^35^. It can significantly discriminate between subgroups of subjects in both clinical and nonclinical settings, and differentiated medical and psychiatric patients from healthy controls^35^. The SQ is a highly sensitive clinimetric index, with good predictive and concurrent validity, and its use in clinical investigations is strongly recommended^35^.

#### Psychological well-being

The Italian version of the semi-structured interview^36^ derived from Psychological Well-Being scales^37^ items (PWB-I) was used to assess psychological well-being and resilience in clinical populations, according to Ryff’s multidimensional model^37^. The original scale has shown good internal consistency^38^ and test–retest reliability^37^. For the purpose of this study, the PWB-I, which includes 18 questions with dichotomous “Yes/No” answers encompassing all the six dimensions of PWB (i.e., self-acceptance, positive relationship with others, purpose in life, environmental mastery, personal growth, autonomy), was used in order to encounter the needs of a busy clinical setting such as CRC screening program.

#### Lifestyle-related behaviors

Lifestyle-related behaviors were assessed with an adaptation of the GOSPEL questionnaire^39,40^, which was specifically designed for the GOSPEL Study^39,40^ in order to overcome the limits of full-scale questionnaires on food frequency and leisure time physical activity that usually are not likely to be suitable to busy real-world clinical setting^39,40^. It has been used in previous studies on patients with medical conditions^10,41,42^. The instrument includes items evaluating the frequencies of physical activity, specific eating habits tailored to maximize detection of dietary variation among Italian adults (i.e., consumption of fruit, vegetables, fish, dairy products, red/processed and white meat), alcohol consumption and tobacco smoking (cigarettes), rated on a 4-point Likert scale (*never/occasionally*; *2/3 times a week; once a day; more than once a day*).

#### Endoscopic outcomes

The endoscopic outcomes were obtained from the Bellaria Hospital Screening center a week after patients’ colonoscopy. Endoscopic outcomes were classified by the gastroenterologist as “negative”, when the colonoscopy did not show any type of lesion, or “positive”, when the colonoscopy showed major endoscopic outcomes (i.e., precancerous lesions such as neoplasms and adenomas) or minor endoscopic outcomes (i.e., hyperplastic polyps, diverticula and hemorrhoids). Among positive endoscopic outcomes, we focused on major diagnoses involving precancerous lesions^43^ that were treated with polypectomy afterwards.

### Data analysis

Data were entered into SPSS for Windows 20.0 (SPSS Inc., Chicago, IL, USA). Descriptive analyses were run for frequencies of socio-demographic, clinical characteristics and lifestyle-related behaviors of the sample. Multivariate analyses of variance using the General Linear Model were performed to test the associations between DCPR-R classification and scores obtained from the dimensional psychological measures (SQ) and the association between DCPR-R diagnoses and psychological well-being (PWB-I) scores. To evaluate the associations between DCPR classification, lifestyle and endoscopic diagnoses, χ^2^-test applied to contingency tables was used, as appropriate. Significance level was set to 0.05, two-tailed.

## Results

Three hundred and sixty patients were approached and asked to join the study. Among them, 210 (58.3%) declined to participate (the main reason was lack of time). One hundred and fifty consecutive participants (41.7%) were enrolled in the study (mean age = 60.90 ± 5.57 years; M = 52%). 16 (10.7%) joined the screening program for the first time, whereas the majority of the sample (N = 134; 89.3%) joined it repeatedly over time. Sociodemographic data are described in Table 1.

**Table 1:** Socio-demographic, clinical characteristics and lifestyle-related behaviors of the sample (N = 150).

	Number	Frequency
**Socio-demographic and clinical characteristics**		
**Sex**		
Male	78	52%
Female	72	48%
**Marital status**		
Single	12	8.0%
Married	110	73.3%
Divorced/widowed	28	18.7%
**Participation to the screening program (every 2 years)**		
First time	16	10.7%
Second time	23	15.3%
Third time	17	11.3%
Fourth time	14	9.3%
Fifth time	25	16.7%
Sixth time	16	10.7%
Seventh time	38	25.3%
Eighth time	1	0.7%
**Colonoscopy**		
Participants who underwent colonoscopy	134	89.3%
Participants who refused colonoscopy	16	10.7%
**Endoscopic outcomes**		
Negative diagnosis at colonoscopy	30	22.4%
Positive diagnosis at colonoscopy	104	77.6%
Adenomas (all treated with Polypectomy)	56	41.8%
Adenoma with Low Grade Dysplasia (LGD)	50	89.3%
Adenoma with High Grade Dysplasia (HGD)	6	10.7%
**Lifestyle-related behaviors**		
**Physical activity**		
Never	76	50.7%
Once/twice a week	31	20.7%
Once a day	18	12%
More than once a day	25	16.7%
**Consumption of vegetables**		
Never	11	7.3%
Once/twice a week	36	24%
Once a day	54	36%
More than once a day	49	32.7%
**Consumption of fruits**		
Never	20	13.3%
Once/twice a week	23	15.3%
Once a day	55	36.7%
More than once a day	52	34.7%
**Consumption of dairy products**		
Never	49	32.7%
Once/twice a week	26	17.3%
Once a day	55	36.7%
More than once a day	20	13.3%
**Consumption of white meat**		
Never	32	21.3%
Once/twice a week	101	67.3%
Once a day	15	10%
More than once a day	2	1.3%
**Consumption of red/processed meat**		
Never	80	53.3%
Once/twice a week	65	43.3%
Once a day	5	3.3%
More than once a day	0	0%
**Consumption of fish**		
Never	89	59.3%
Once/twice a week	54	36%
Once a day	5	3.3%
More than once a day	2	1.3%
**Consumption of alcohol**		
Never	145	96.7%
Once/twice a week	2	1.3%
Once a day	2	1.3%
More than once a day	1	0.7%
**Smoking habit (cigarettes)**		
Never	119	79.3%
Once/twice a week	0	0%
Once a day	0	0%
More than once a day	31	20.7%

**Figure 1:**
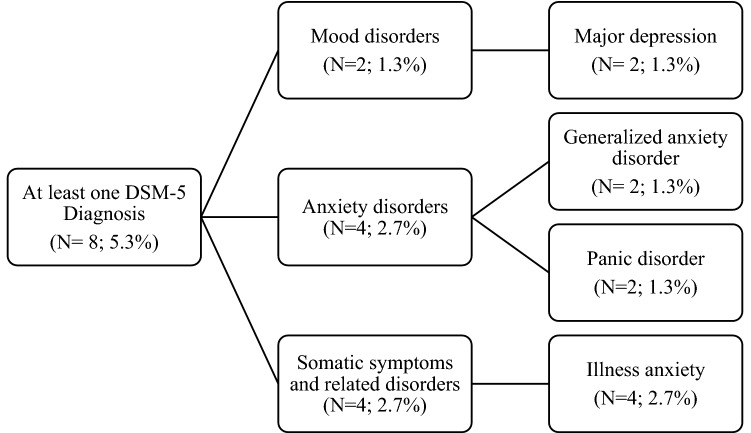
Psychiatric diagnoses (DSM-5).

Regarding the first aim of the present study, eight participants (5.3%) reported at least one DSM-5 diagnosis (panic disorder = 2, generalized anxiety disorder = 2, illness anxiety disorder = 2, major depression = 2) (Fig. 1). Only 2 received more than one DSM-5 diagnosis (panic disorder and illness anxiety disorder; major depression and illness anxiety disorder).

**Figure 2:**
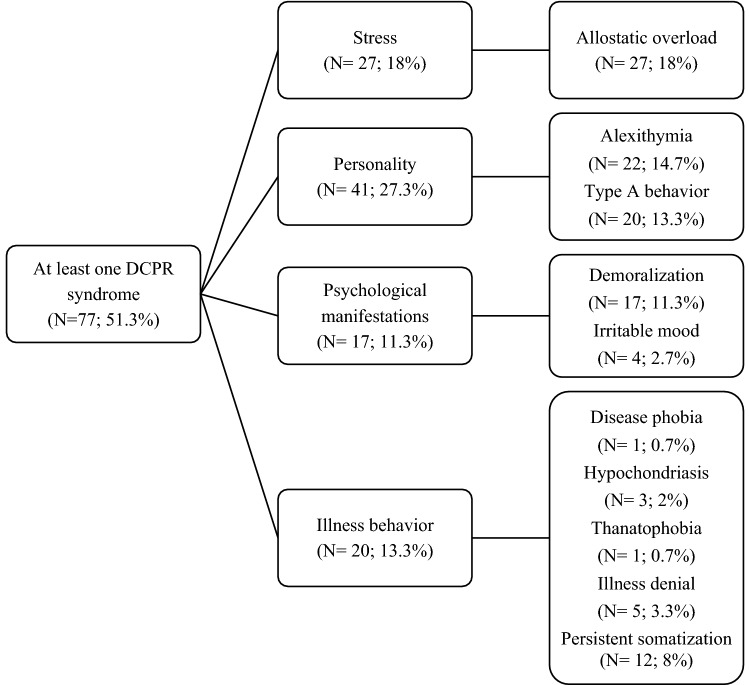
Psychosomatic syndromes (DCPR-R).

Seventy-seven subjects (51.3%) presented with at least one psychosomatic syndrome according to DCPR-R (Fig. 2). Among these, 19 (12.6%) presented with more than one DCPR-R diagnosis. 5 patients (3.3%) reported a comorbidity between DCPR-R and DSM-5. The most frequent DCPR-R diagnoses were allostatic overload (N = 27; 18%), alexithymia (N = 22; 14.7%), type A behavior (N = 20; 13.3%) and demoralization (N = 17; 11.3%) (Fig. 2).

Compared to general population^34^, the overall sample did not self-report a higher level of psychological distress, as assessed by the four scales of SQ. However, the subgroup of patients meeting criteria for DCPR-R syndromes showed significantly higher scores of distress in all the four scales of Symptom Questionnaire (all *p* < 0.001) (Table 2), compared to non-cases. In addition, patients affected by DCPR-R diagnoses showed significantly lower scores in almost all the dimensions of PWB-I: self-acceptance (*p* < 0.001), positive relations with others (*p* < 0.001), purpose in life (*p* = 0.001) and environmental mastery (*p* = 0.005) (Table 2).

**Table 2:** Differences on SQ and PWB mean scores of participants with at least one DCPR syndrome compared with non-cases.

	DCPR (+) (N = 77) mean ± SD	DCPR (−) (N = 73) mean ± SD	F	*df*	*p*
SQ anxiety	7.34 ± 4.73	4.25 ± 3.47	20.629	1	< 0.001
SQ depression	5.94 ± 4.77	2.95 ± 2.51	22.696	1	< 0.001
SQ somatization	7.62 ± 4.02	5.11 ± 3.62	16.156	1	< 0.001
SQ hostility/irritability	4.95 ± 3.52	2.71 ± 2.50	19.962	1	< 0.001
PWB-I self-acceptance	2.40 ± 0.98	2.88 ± 0.37	15.128	1	< 0.001
PWB-I positive relations with others	2.26 ± 0.92	2.73 ± 0.58	13.499	1	< 0.001
PWB-I purpose in life	2.13 ± 0.92	2.58 ± 0.64	11.642	1	0.001
PWB-I environmental mastery	2.48 ± 0.88	2.81 ± 0.46	7.990	1	0.005
PWB-I personal growth	2.58 ± 0.70	2.73 ± 0.51	2.014	1	0.158
PWB-I autonomy	1.99 ± 0.90	2.19 ± 0.84	2.071	1	0.152

(+) Syndrome present, (−) Syndrome absent, *DCPR* diagnostic criteria for psychosomatic research, *PWB-I* psychological well-being interview, *SQ* Symptom Questionnaire.

Concerning unhealthy lifestyle-related behaviors (Table 1), 76 participants (50.7%) never performed any physical activity, whereas only 31 (20.7%) did it at least once or twice a week. Moreover, 11 (7.3%) never ate vegetables, whereas only 36 (24%) did it at least once or twice a week. On the same vein, 20 participants (13.3%) never consumed fruits and only 23 (15.3%) did it at least once or twice a week. Half of the sample never ate dairy products (N = 49; 32.7%) or consumed them only once or twice a week (N = 26; 17.3%). 5 (3.3%) participants reported to eat red or processed meat once a day, whereas the majority of the sample (N = 89; 59.3%) never ate fish. Almost all the subjects (N = 145; 96.7%) declared to never drink alcohol. Finally, 31 participants (20.7%) were smokers.

With regard to the second aim of the present study, specific associations between DCPR-R syndromes and lifestyle-related behaviors were found (Table 3). In particular, participants who were diagnosed with allostatic overload (N = 27), were significantly more likely to smoke cigarettes than who did not present with the same diagnosis (40.7% *versus* 16.3%; χ^2^ = 8.093; df = 1; *p* < 0.01) (Table 3). Participants diagnosed with persistent somatization, compared with non-cases, were significantly less likely to eat fruit (50% versus 10.1% never ate fruit; χ^2^ = 15.344; df = 3; *p* < 0.01) and dairy products (50% versus 31.2% never ate dairy products; χ^2^ = 10.250; df = 3; *p* < 0.05) (*Table **3*).

**Table 3:** Associations between DCPR allostatic overload, persistent somatization and lifestyle-related habits.

	DCPR allostatic overload (+) (N = 27) *N* (%)				DCPR allostatic overload (−) (N = 123) *N* (%)				*χ*^*2*^	*df*	*p*	DCRP persistent somatization (+) (N = 12) *N* (%)				DCRP persistent somatization (−) (N = 138) *N* (%)				*χ*^*2*^	*df*	*p*
Lifestyle	Never	Once/twice a week	Once a day	More than once a day	Never	Once/twice a week	Once a day	More than once a day				Never	Once/twice a week	Once a day	More than once a day	Never	Once/twice a week	Once a day	More than once a day			
Physical activity	16 (59.3%)	6 (22.2%)	4 (14.8%)	1 (3.7%)	60 (48.8%)	25 (20.3%)	14 (11.4%)	24 (19.5%)	4.056	3	0.256	6 (50%)	2 (16.7%)	3 (25%)	1 (8.3%)	70 (50.7%)	29 (21%)	15 (10.9%)	24 (17.4%)	2.483	3	0.478
Consumption of vegetables	1 (3.7%)	9 (33.3%)	9 (33.3%)	8 (29.6%)	10 (8.1%)	27 (22%)	45 (36.6%)	41 (33.3%)	1.945	3	0.584	1 (8.3%)	2 (16.7%)	7 (58.3%)	2 (16.7%)	10 (7.2%)	34 (24.6%)	47 (34.1%)	47 (34.1%)	3.139	3	0.371
Consumption of white meat	9 (33.3%)	14 (51.9%)	4 (14.8%)	0 (0%)	23 (18.7%)	87 (70.7%)	11 (8.9%)	2 (1.6%)	4.597	3	0.204	5 (41.7%)	5 (41.7%)	2 (16.7%)	0 (0%)	27 (19.6%)	96 (69.6%)	13 (9.4%)	2 (1.4%)	4.558	3	0.207
Consumption of red/processed meat	18 (66.7%)	9 (33.3%)	0 (0%)	0 (0%)	62 (50.4%)	56 (45.5%)	5 (4.1%)	0 (0%)	2.955	2	0.228	9 (75%)	3 (25%)	0 (0%)	0 (0%)	71 (51.4%)	62 (44.9%)	5 (3.6%)	0 (0%)	2.595	2	0.273
Consumption of fruits	4 (14.8%)	4 (14.8%)	8 (29.6%)	11 (40.7%)	16 (13%)	19 (15.4%)	47 (38.2%)	41 (33.3%)	0.855	3	0.836	6 (50%)	1 (8.3%)	2 (16.7%)	3 (25%)	14 (10.1%)	22 (15.9%)	53 (38.4%)	49 (35.5%)	15.344	3	0.002
Consumption of dairy products	7 (25.9%)	4 (14.8%)	11 (40.7%)	5 (18.5%)	42 (34.1%)	22 (17.9%)	44 (35.8%)	15 (12.2%)	1.391	3	0.708	6 (50%)	5 (41.7%)	1 (8.3%)	0 (0%)	43 (31.2%)	21 (15.2%)	54 (39.1%)	20 (14.5%)	10.250	3	0.017
Consumption of fish	16 (59.3%)	9 (33.3%)	1 (3.7%)	1 (3.7%)	73 (59.3%)	45 (36.6%)	4 (3.3%)	1 (0.8%)	1.466	3	0.690	7 (58.3%)	4 (33.3%)	1 (8.3%)	0 (0%)	82 (59.4%)	50 (36.2%)	4 (2.9%)	2 (1.4%)	1.180	3	0.758
Consumption of alcohol	27 (100%)	0 (0%)	0 (0%)	0 (0%)	118 (95.9%)	2 (1.6%)	2 (1.6%)	1 (0.8%)	1.135	3	0.769	12 (100%)	0 (0%)	0 (0%)	0 (0%)	133 (96.4%)	2 (1.4%)	2 (1.4%)	1 (0.7%)	0.450	3	0.930
Smoking habit	16 (59.3%)	0 (0%)	0 (0%)	11 (40.7%)	103 (83.7%)	0 (0%)	0 (0%)	20 (16.3%)	8.093	1	0.004	9 (75%)	0 (0%)	0 (0%)	3 (25%)	110 (79.7%)	0 (0%)	0 (0%)	28 (20.3%)	0.149	1	0.699

(+) Syndrome present; (−) Syndrome absent, *DCPR* diagnostic criteria for psychosomatic research.

Sixteen participants (10.7%) refused to undergo the colonoscopy after the psychological interview (mainly for lack of time or because they had already booked the medical exam privately). Among the 134 CRC-screening participants who underwent the colonoscopy, 104 (69.3%) got a positive endoscopic diagnosis. Among them, no one presented a neoplasm, whereas 56 (53.8%) showed adenomas (i.e., Low Grade Dysplasia—LGD = 50; High Grade Dysplasia—HGD = 6) treated with polypectomy afterwards (Table 1).

As to the associations between lifestyle-related behaviors, psychosocial characteristics and endoscopic outcomes, no difference concerning lifestyle behaviors between positive versus negative diagnoses was found. Participants with adenomas treated with polypectomy reported a significantly higher frequency of DCPR-R irritable mood (Table 4). Specifically, all the participants who satisfied criteria for irritable mood were diagnosed with adenomas after colonoscopy and underwent polypectomy (χ^2^ = 5.743; df = 1; p < 0.05) (Table 4). No difference in the distribution of adenomas according to sex was found, neither between younger (≤ 59 years old) and older (> 59 years old) participants.

**Table 4:** Association between adenomas and psychosomatic syndromes (DCPR).

DCPR syndrome	Adenoma (+) (N = 56) *N* (%)	Adenoma (−) (N = 78) *N* (%)	*χ*^*2*^	df	*p*
Allostatic overload	7 (28%)	18 (72%)	2.403	1	0.121
Alexithymia	10 (47.6%)	11 (52.4%)	0.348	1	0.555
Type A behavior	8 (42.1%)	11 (57.9%)	0.001	1	0.976
Disease phobia	1 (100%)	0 (0%)	1.403	1	0.236
Hypochondriasis	1 (33.3%)	2 (66.7%)	0.090	1	0.764
Thanatophobia	1 (100%)	0 (0%)	1.403	1	0.236
Illness denial	3 (75%)	1 (25%)	1.869	1	0.172
Persistent Somatization	4 (40%)	6 (60%)	0.014	1	0.905
Demoralization	6 (40%)	9 (60%)	0.022	1	0.881
Irritable mood	4 (100%)	0 (0%)	5.743	1	0.017

*DCPR* Diagnostic Criteria for Psychosomatic Research, (+) Presence of adenomas (treated with polypectomy), (−) Absence of adenomas.

## Discussion

In a clinical context of secondary prevention addressing asymptomatic patients who had positive fecal occult blood test, a comprehensive psychosomatic assessment based on clinimetric principles can provide relevant clinical information that—relying on traditional psychiatric taxonomy only—would have gone undetected. Indeed, determination of psychiatric disorders according to DSM diagnostic criteria^21^ is essentially based on a fixed number of key symptoms that have to be satisfied. However, setting a clinical threshold merely on the basis of these criteria may be tricky in patients who do not manifest somatic symptoms, and may miss important clinical information. In the present investigation, an innovative approach to the assessment of psychological and psychosomatic profile, together with lifestyle habits, was attempted. Indeed, the results of the present investigation found that more than half of the participants in CRC screening showed at least one DCPR psychosomatic syndrome, particularly allostatic overload, associated with high psychological distress, impaired psychological well-being, unhealthy lifestyle and colorectal precancerous lesions. On the contrary, only a small percentage of patients (5.3%) met criteria for a DSM-5 diagnosis.

Literature shows a paucity of study aimed to identify psychosocial distress, psychological well-being, and lifestyle behaviors in participants at the secondary prevention program of CRC screening^9,15^. Concerning psychosocial distress, the present investigation found allostatic overload, alexithymia, type A behavior and demoralization as the most frequent DCPR diagnoses. A high percentage of DCPR syndromes in gastroenterology setting has been found in patients with functional gastrointestinal disorders^44^. However, DCPR diagnoses in asymptomatic participants at the CRC screening program did never emerge before in the literature. Otherwise, studies in literature reported mostly mixed results concerning worry and anxiety among participants after receiving the result of positive fecal occulted blood test^45,46^. The findings of our investigation support the growing body of literature showing that DCPR criteria provide a better explanatory model for clinical phenomena in medical settings, which are not detected by traditional psychiatric nosography^32,47^. Moreover, Ferrari et al.^48^ underlined the clinical utility of DCPR in revealing patients with high psychological distress. Indeed, despite the fact that our sample did not self-report a higher level of psychological distress compared to general population^34^, DCPR system allowed the identification of a vulnerable subgroup of patients showing significantly higher scores of anxiety, depression, somatization and hostility-irritability symptoms (SQ), as well as lower levels of psychological well-being at PWB-I (i.e., self-acceptance, positive relations with others, purpose in life and environmental mastery), compared to patients without psychosomatic syndromes. The higher scores of psychological distress at SQ, as well as the lower psychological well-being at PWB-I, which have been found in participants with DCPR syndromes seem to support the literature showing that psychological distress reported by participants who resulted positive at CRC screening tests, might be related to pre-existent psychological conditions rather than worry about colonoscopy and screening result itself^49–51^.

Concerning lifestyle-related behaviors, we found impressive results in our total sample. Indeed, participants reported no or poor physical activity (over 70% of the sample), poor consumption of vegetables (over 30%), fruit (one quarter), dairy products (half), whereas nearly 60% reported no consumption of fish. Findings of the present investigation suggest a possible role of psychosomatic syndromes. Indeed, significant associations between DCPR psychosomatic syndromes, in particular persistent somatization and allostatic overload, and unhealthy behaviors have been found. Persistent somatization seems to be associated to a lower consumption of dairy products and fruit. This finding is in line with few studies in literature. Trabal et al.^52^ found that the most prevalent restrictions in patients with chronic fatigue syndrome, which is characterized by enduring and long-term somatic symptoms, were related to dairy products and gluten-containing grains. Similarly, Goedendorp et al.^53^ found that 70% of patients with chronic fatigue syndrome had unhealthy fat, fruit and vegetable intake. The same has been found for patients with fibromyalgia who had significantly lower mean consumption of different products such as fruits^54^. On the same line, in our study allostatic overload, which reflects the cumulative effects of stressful experiences in daily life^31^ and has been shown to affect clinical course and survival in cardiac patients^28,55^, seems to be associated with tobacco smoking. This finding is in line with the results of Sotos-Prieto and colleagues’ study^56^, in which participants with a chronic stress situation were more likely to be smokers. This risky association (i.e., allostatic overload and smoke habit) should be taken particularly into account given its increased risk for cardiovascular health. The role of DCPR irritability found in the present work is noteworthy. The totality of participants who met criteria for DCPR irritable mood had precancerous lesions (all LGD adenomas), treated with polypectomy. The presence of DCPR irritable mood was reported in patients diagnosed with different types of cancer by Mangelli and colleagues in 2006^57^. Results from White and colleagues’ study^58^ suggested that negative affect might play a small role in colorectal cancer, whereas anger control might not. Specifically, the authors advocated that the experience of negative emotions, rather than their repression or control, seems to be associated with colorectal cancer risk. However, White et al.^58^ used only self-rating questionnaires in order to assess both anger control and negative affect. The use of a self-rating assessment in psychosomatic research presents well-known limits (i.e., patients may feel uncomfortable to complete the survey, may not understand the questions or, when asked to quantify psychological distress, provide confusing information). We have tried to overcome these limits in our work providing a comprehensive psychosomatic assessment^16^ that includes both self-rating questionnaires and well-structured clinical interviews. There is the need of further prospective studies on evaluating the role of anger/irritability in early onset of colorectal cancer.

Finally, concerning the association between lifestyle behaviors and major endoscopic outcomes or precancerous lesions, we expected that participants who had adenomas treated with polypectomy would have shown worse lifestyle-related habits, compared with those with minor endoscopic outcomes, as reported in the literature^59^. In contrast to our expectations, however, we did not find any difference between major and minor endoscopic outcomes concerning diet, smoking habits or physical activity. A possible explanation of this finding could be linked to the small sample size and the fact that in our sample there were no cases of cancer. On the other hand, at this screening stage, it seems that DCPR system could be more sensitive in sub-grouping populations at high risk for cancer either for the presence of adenomas or unhealthy behaviors, underlining its clinical utility.

The present investigation presents some limitations, such as the small sample size, the cross‐sectional study design (despite this, we were able to include many important factors in the comprehensive psychosomatic assessment), the absence of a control group and the fact that 16 participants refused colonoscopy. This latter aspect, however, reflects previous literature showing that not all participants at CRC primary screening complete the procedure and undergo secondary screening of colonoscopy^8^. Future studies should investigate if psychosomatic distress evaluated by DCPR might play a role in the decision to adhere to CRC screening program.

## Conclusions

The present findings offer two main clinical implications, one concerning the innovative approach to the assessment used in this study and the other regarding specific suggestions for future preventive programs. On one hand, indeed, this investigation showed the clinical utility of a comprehensive psychosomatic approach based on clinimetric principles, including both observer- and self-rated measures^60^, that provided clinical information for a substantial number of patients referred to CRC screening who do not satisfy DSM-5 classification criteria and yet present with high levels of stress and psychological distress, impaired well-being, unhealthy lifestyle, and risk for CRC. On the other hand, given that the modification of unhealthy lifestyle behaviors could be moderated by the presence of psychological distress and psychosomatic suffering^10^, early detection of specific DCPR syndromes such as irritable mood, allostatic overload and persistent somatization, in association with poor lifestyle habits, has important implications not only for mental illness^61,62^, but also for secondary prevention of CRC. In response to the growing attention given to the need to promote physical health in persons with medical and mental illnesses^62^, as well as to the difficulties in implementing major lifestyle changes or widespread primary prevention strategies to decrease CRC risk^4^, the results of the present study might indicate a road to the practice of “preventive” or lifestyle medicine at CRC screening program. Indeed, tailored interventions addressing specific psychosomatic profiles (such as those characterized by difficulties in managing stress, irritability and somatic manifestations of psychological distress), might have an impact on those factors that have been found to hinder lifestyle changes^10^ and possibly improve biochemical correlates^63^. Future research should investigate the potential of programs for healthy lifestyle secondary prevention addressing psychosomatic suffering, by extending them to other kinds of screening programs and focusing on younger persons who are at higher risk for developing CRC cancer^64^, in order to implement them in policy-making, especially in the pandemic era in which an overall worsening of the lifestyle has spread^65,66^.

## Data Availability

The dataset generated and analyzed during the current study is available from the corresponding author (chiara.rafanelli@unibo.it) upon reasonable request.
